# Supplementary material for: Brain microRNAs among social and solitary bees
Source: R Soc Open Sci. 2020 Jul 8;7(7):200517. doi: 10.1098/rsos.200517 (PMC7428247; doi:10.1098/rsos.200517)
Supplement: SUPPLEMENTARY MATERIALS [file rsos200517supp1.pdf]

SUPPLEMENTARY MATERIALS

Brain microRNAs among social and solitary bees

Karen M. Kapheim, Beryl M. Jones, Eirik Søvik, Eckart Stolle, Robert M. Waterhouse, Guy Bloch, Yehuda Ben-Shahar

Table S1. Genome assemblies used for alignment of small RNA sequences or genome scans for known small RNAs. The last six species were used only for genome scans.

Species	Genome assembly	Reference
Apis mellifera	Amel v4.5	Elsik et al. 2014
Bombus impatiens	Bimp v2.0	Sadd et al. 2015
Bombus terrestris	Bter v1.0	
Megalopta genalis	Mgen v1.0	Kapheim et al., 2020
Megachile rotundata	Mrot v1.0	Kapheim et al. 2015
Nomia melanderi	Nmel v1.0	Kapheim et al. 2019
Apis florea	Aflo v1.0	Baylor College of Medicine, unpublished (NCBI PRJNA45871)
Dufourea novaeangliae	Dnov v1.0	Kapheim et al. 2015
Eufriesea mexicana	Emex v1.0	Kapheim et al. 2015
Habropoda laboriosa	Hlab v1.0	Kapheim et al. 2015
Lasioglossum albipes	Lalbi v2	Kocher et al. 2013
Melipona quadrifasciata	Mqua v1.0	Kapheim et al. 2015

Table S2. Sample acquisition and library preparation methods. Samples were collected into liquid nitrogen and stored at -80 °C until dissection. All libraries were prepared from RNA isolation from whole brain tissue. All libraries were sequenced for 51 cycles on a HiSeq 2500. *B. impatiens*, *M. genalis*, *N. melanderi* libraries were pooled and sequenced in one lane. *A. mellifera*, *B. terrestris*, and *M. rotundata* libraries were pooled and sequenced in one lane.

Species	Collection	Sample type	RNA Isolation	RNA Quality Assessment	Sequencing Center	Library Prep	# reads	Reads mapped to genome
Bombus impatiens	Commercial colony (BioBest, Romulus, MI, USA)	Worker	mirVana miRNA Isolation kit with phenol (Ambion)	TapeStation (Agilent) – USU Center for Integrated Biosystems	University of Illinois Roy J. Carver Biotech Center	Illumina TruSeq Small RNA Sample Preparation kit	18,173,120	11,147,865 (61.3%)
Megalopta genalis	Barro Colorado Island, Panama[¥]	Lab-reared female					17,273,381	14,459,264 (83.7%)
Nomia melanderi	Touchet, WA, USA	Reproductive female					21,916,316	16,681,729 (76.1%)
Apis mellifera	Urbana-Champaign, IL; Tyson Research Station, St. Louis, MO, USA	Worker	TRIzol reagent (Thermo Fisher Scientific)	Bioanalyzer (Agilent) - Washington University Genome Technologies Access Center	Washington University Genome Tech Access Center	Illumina TruSeq, Clontech SMARTer small RNA library kit	12,793,471	7,638,748 (59.7%)
Bombus terrestris	Commercial colony (Pollination Services Yad-Mordechai, Kibbutz Yad-Mordechai, Israel)	Worker					16,270,644	11,205,739 (68.9%)
Megachile rotundata	Logan, UT, USA	Reproductive female					19,160,796	15,357,334 (80.1%)

[¥]*M. genalis* samples were exported under permit SEX/A-37-15

Table S3. Known microRNAs used in miRDeep2 microRNA detection protocol.

Species	Source	Reference
Apis mellifera	miRBase v21	Kozomara and Griffiths-Jones 2014
Drosophila melanogaster		
Nasonia vitripennis		
Tribolium castenum		
Bombyx mori		
Apis mellifera	Small RNA sequencing	Ashby et al. 2016 (Table S1)

Table S4. Gene models used for localization of microRNAs and predicted target analysis.

Species	Genome annotation	Reference
Apis mellifera	Amel OGS v3.2	Elsik et al. 2014
Bombus impatiens	Bimp OGS v1.0	Elsik et al. 2016
Bombus terrestris	Bter v1.3	Sadd et al. 2015
Megalopta genalis	Mgen v1.0	Kapheim et al. 2020
Megachile rotundata	Mrot v1.1	Kapheim et al. 2015
Nomia melanderi	Nmel v1.0	Kapheim et al. 2019

Table S5 (separate file). Enrichment results for predicted targets of lineage-specific miRs and "social genes". Includes gene lists, conversion lists based on reciprocal blastp results, and overlap test statistics. Column head descriptions are in 'ColumnDetails' sheet.

Table S6 (separate file). Results from genome scans for miRNA seed matches in Rfam. Descriptions for each sheet and column header are provided in the 'Metadata' sheet.

Table S7 (separate file). Final miRNA sets for each species. Descriptions for each sheet and column header are provided in the 'Metadata' sheet.

Table S8 (separate file). Predicted targets and orthogroup ages of lineage-specific microRNAs in each species. Descriptions for each sheet and column header are provided in the 'Metadata' sheet.

Figure S1 (separate file). Predicted targets of lineage-specific miRNAs in relation to social behavior. Genes that are both predicted targets of lineage-specific miRNAs and genes with differential expression in a social context (solid outlines) or genes under selection (dashed outlines) are represented by overlapping circles for each study and species. Numbers of lineage-specific miRNA targets are given for each species. Colors indicate different studies. Overlaps not significantly different from random (representation factor, RF=1) are unlabeled, while significant over- or under-enrichments are marked with asterisks with RF and p-value as indicated.

REFERENCES

Ashby R, Forêt S, Searle I, Maleszka R. 2016. MicroRNAs in honey bee caste determination. Sci. Rep. 6:1–15.

Elsik CG, Tayal A, Diesh CM, Unni DR, Emery ML, Nguyen HN, Hagen DE. 2016. Hymenoptera Genome Database: integrating genome annotations in HymenopteraMine. Nucleic Acids Res. 44:D793–D800.

Elsik CG, Worley KC, Bennett AK, Beye M, Camara F, Childers CP, de Graaf DC, Debyser G, Deng J, Devreese B, et al. 2014. Finding the missing honey bee genes: Lessons learned from a genome upgrade. BMC Genomics 15:1–29.

Kapheim KM, Pan H, Li C, Blatti C, Harpur BA, Ioannidis P, Jones BM, Kent CF, Ruzzante L, Sloofman L, et al. 2019. Draft genome assembly and population genetics of an agricultural pollinator, the solitary alkali bee (Halictidae: *Nomia melanderi*). G3. 9:625–634.

Kapheim KM, Pan H, Li C, Salzberg SL, Puiu D, Magoc T, Robertson HM, Hudson ME, Venkat A, Fischman BJ, et al. 2015. Genomic signatures of evolutionary transitions from solitary to group living. Science 348:24–32.

Kapheim KM, Jones BM, Pan H, Harpur BA, Kent CF, Zayed A, Ioannidis P, Waterhouse RW, Kingwell C, Stolle E, Avalos A, Zhang G, McMillan WO, Wcislo WT. 2020. Developmental plasticity shapes the genomic signatures of eusociality. *Proc Nat Acad Sci*. 202000344; (doi:10.1073/pnas.2000344117)

Kocher SD, Li C, Yang W, Tan H, Yi S V, Yang X, Hoekstra HE, Zhang G, Pierce NE, Yu DW. 2013. The draft genome of a socially polymorphic halictid bee, *Lasioglossum albipes*. Genome Biol. 14:R142.

Kozomara A, Griffiths-Jones S. 2014. miRBase: annotating high confidence microRNAs using deep sequencing data. Nucleic Acids Res 42:D68-73.

Sadd BM, Barribeau SM, Bloch G, de Graaf DC, Dearden P, Elsik CG, Gadau J, Grimmelikhuijzen CJ, Hasselmann M, Lozier JD, et al. 2015. The genomes of two key bumblebee species with primitive eusocial organization. Genome Biol. 16:76.
