# Supplementary figures and images for: Brain microRNAs among social and solitary bees
Source: R Soc Open Sci. 2020 Jul 8;7(7):200517. doi: 10.1098/rsos.200517 (PMC7428247; doi:10.1098/rsos.200517)

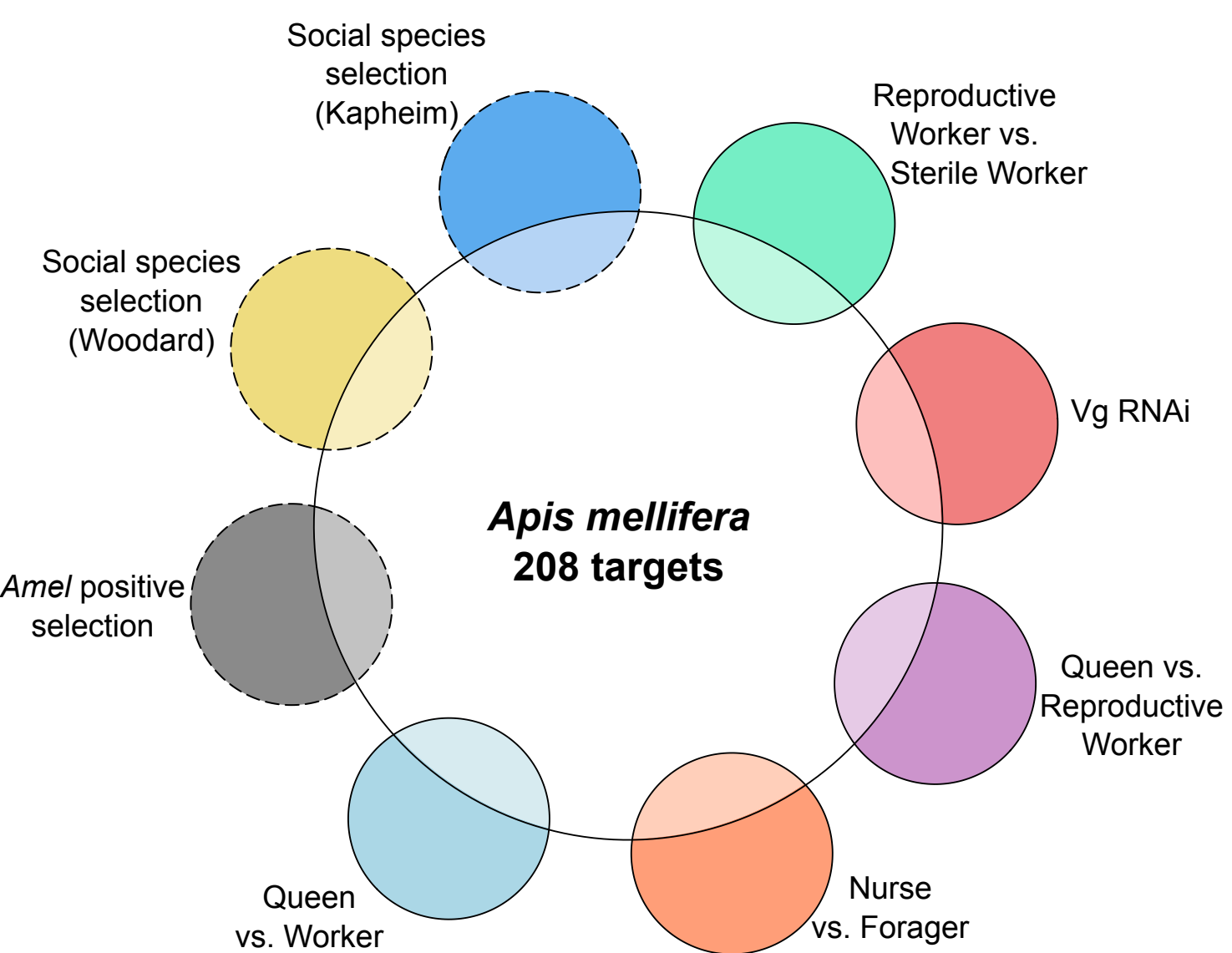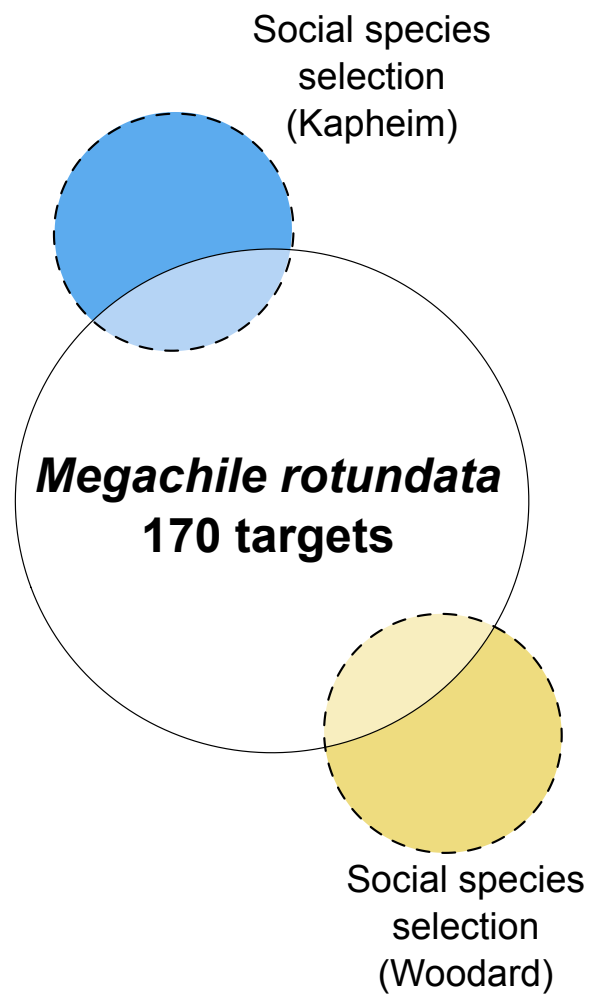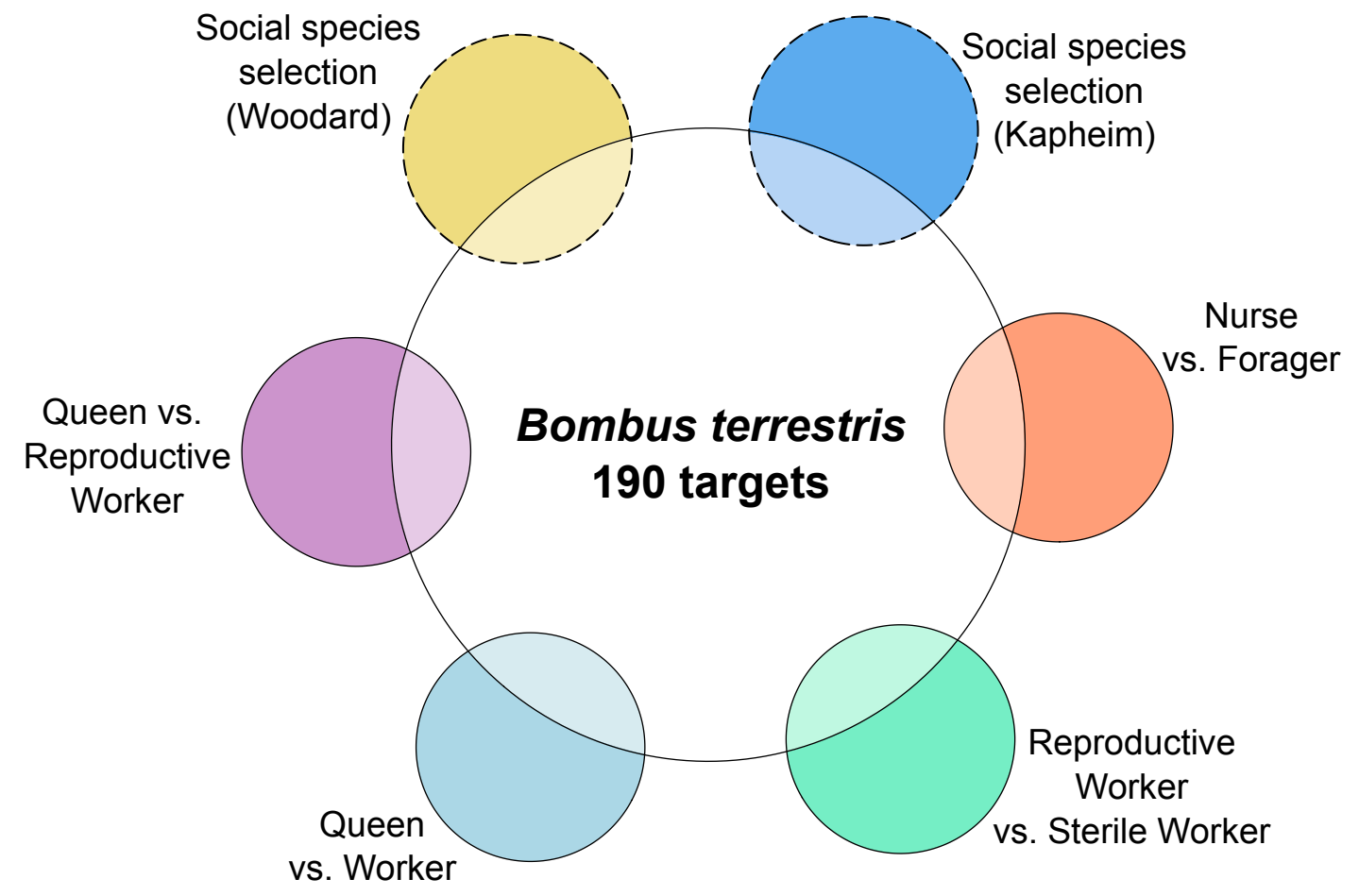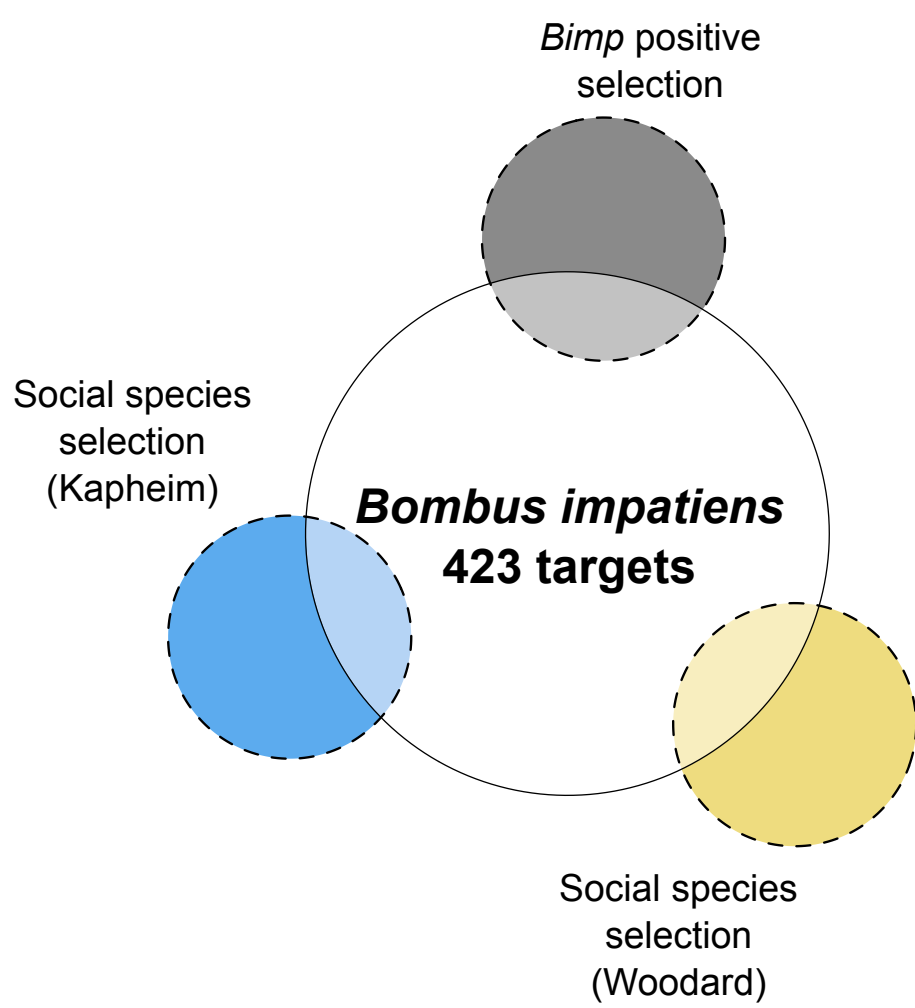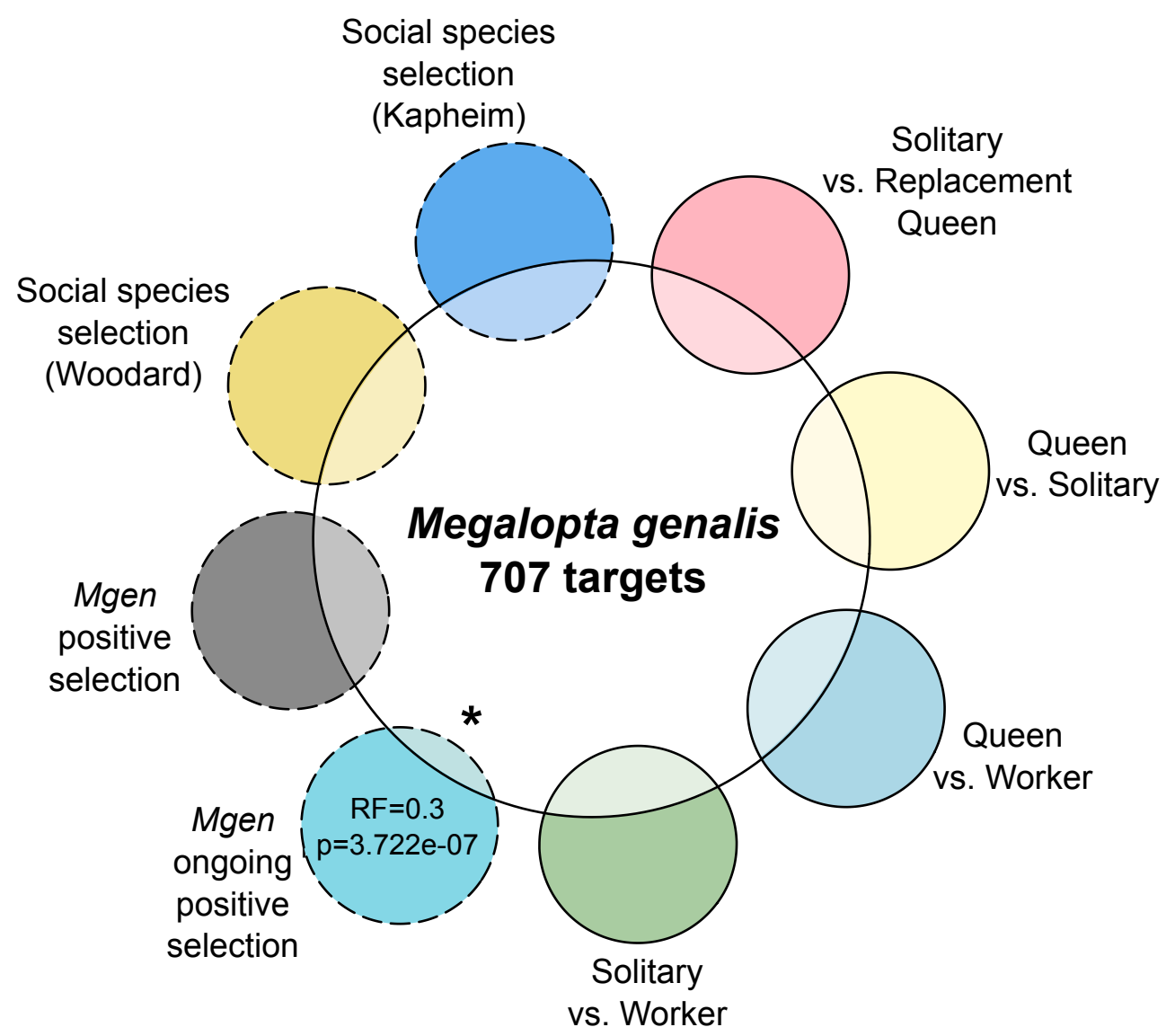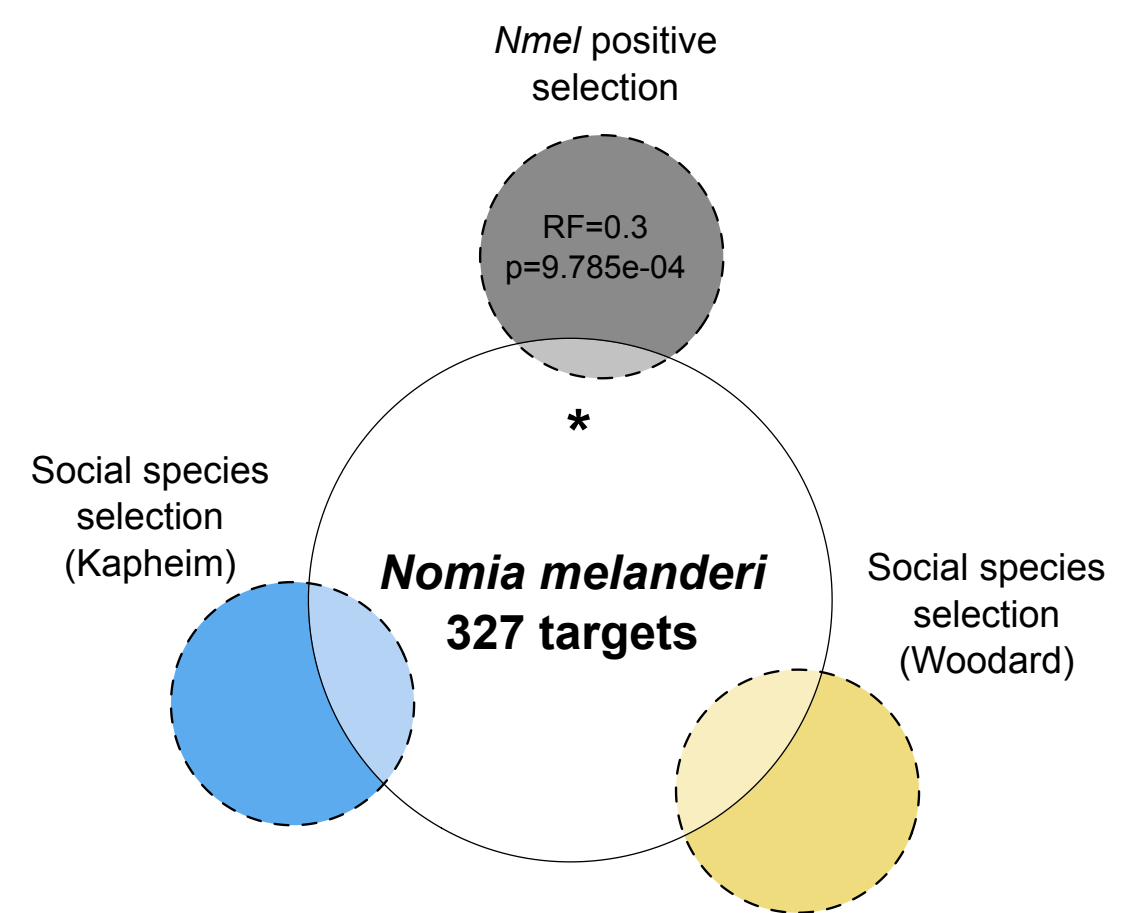

Supplement: Figure S1 [file rsos200517supp6.pdf]
